# Immunoassay for serodiagnosis of Zika virus infection based on time-resolved Förster resonance energy transfer

**DOI:** 10.1371/journal.pone.0219474

**Published:** 2019-07-23

**Authors:** Lauri Kareinen, Satu Hepojoki, Eili Huhtamo, Essi M. Korhonen, Jonas Schmidt-Chanasit, Klaus Hedman, Jussi Hepojoki, Olli Vapalahti

**Affiliations:** 1 University of Helsinki, Medicum, Department of Virology, Helsinki, Finland; 2 Institute of Veterinary Pathology, Vetsuisse Faculty, University of Zürich, Zürich, Switzerland; 3 Bernhard Nocht Institute for Tropical Medicine, Hamburg, Germany; 4 German Centre for Infection Research (DZIF), Partner Site Hamburg-Luebeck-Borstel, Hamburg, Germany; 5 Helsinki University Hospital, HUSLAB, Helsinki, Finland; International Centre for Genetic Engineering and Biotechnology, ITALY

## Abstract

Zika virus (ZIKV) is a mosquito-borne pathogen causing a febrile illness with arthralgia, conjunctivitis and rash. The complications include Guillain-Barré syndrome, congenital brain and other abnormalities and miscarriage. The serodiagnosis of ZIKV infection is hampered by cross-reactivity with other members of the *Flavivirus* family, notably dengue (DENV). This report describes a novel serological platform for the diagnosis of ZIKV infection. The approach utilizes time-resolved Förster resonance energy transfer (TR-FRET) elicited by two chromophore-labeled proteins (a ZIKV antigen and a super-antigen) simultaneously binding to a given antibody molecule. The antigen used in the assay is ZIKV non-structural protein 1 (NS1) and the super-antigen is bacterial protein L. Three assay variants were developed: the first measuring all anti-ZIKV-NS1 antibodies (LFRET), the second measuring IgM and IgA (acute-LFRET) and the third measuring IgG (immunity-LFRET). The assays were evaluated with a panel of samples from clinical ZIKV cases in travelers (n = 25) and seronegative (n = 24) samples. DENV (n = 38), yellow fever (n = 16) and tick-borne-encephalitis (n = 20) seropositive samples were examined for assessment of flavivirus cross-reactivity. The diagnostic sensitivities of the respective LFRET assays were 92%, 100% and 83%, and the diagnostic specificities 88%, 95% and 100% for LFRET, acute-LFRET and immunity-LFRET. Furthermore, we evaluated the assays against a widely-used commercial ELISA. In conclusion, the new FRET-based serological approaches based on NS1 protein are applicable to diagnosing zika virus infections in travelers and differentiating them from other flavivirus infections.

## Introduction

The rapid and accurate diagnosis of the causative agent is the first step in effective management of infectious disease. Many agents have similar symptoms and without clear laboratory diagnostics the therapeutic interventions can be easily misdirected. A case in point is the Zika virus epidemic, where several viruses circulate in the same areas with similar outward symptoms. The Zika virus (ZIKV) is a mosquito-borne pathogen initially isolated in 1947 in Uganda. Since then only sporadic cases of ZIKV infection with mild clinical manifestations were reported in Africa and Southeast Asia [1]. However, in 2007 an outbreak of febrile illness associated with rash and arthralgia occurred in the Yap island of Micronesia. The causative agent was found to be ZIKV, and retrospective serological diagnostics demonstrated 73% of the residents to be ZIKV seropositive [1]. During the following years ZIKV gradually spread throughout the Micronesian archipelago and made its way to the western hemisphere, with the first outbreak reported in Bahia, Brazil, 2015 [2]. Thereafter, ZIKV advanced rapidly across the South American continent, most severely affecting Brazil with over 220,000 clinically confirmed cases by January 2018 [3].

The clinical picture of ZIKV primary infection tends to be mild including rash, headache, conjunctivitis, arthralgia, myalgia and occasional fever [1]. The symptoms are usually self-limited with an average duration of three to six days, or the infection may be asymptomatic [1]. On the other hand, ZIKV infection can end up with severe neurological sequelae such as Guillain-Barré syndrome [4]. Furthermore, clinical and epidemiological studies have confirmed a causal relationship between ZIKV infection during pregnancy and severe congenital abnormalities, such as microcephaly [5, 6].

ZIKV belongs to the family *Flaviviridae* along with several other important arboviral pathogens, such as dengue virus (DENV), yellow fever virus (YFV), tick-borne encephalitis virus (TBEV) and West Nile virus (WNV). Flaviviruses have a single-stranded, positive-sense RNA genome of ~11 kilobases (kb). The genome has a single open reading frame encoding three structural and seven non-structural proteins. The structural proteins (capsid C, envelope E and matrix M) form the flavivirus particle while the non-structural proteins (NS1, NS2a, NS2b, NS3, NS4a, NS4b and NS5) participate in virus replication. Both also play a part in immune evasion. The E protein is the principal target for neutralizing antibody response [7]. However, a notable portion of the E protein epitopes are shared across different flavivirus species, providing a major source of serodiagnostic cross-reactivity [8]. In particular, the DENV E protein is antigenically so closely related to that of ZIKV that a ZIKV-DENV super serogroup has been suggested [8]. The NS1 has a wide variety of functions and is relatively conserved also among flaviviruses [9]. Inside the infected cells, NS1 is present as a dimer and assists in virus replication and suppression of the interferon response [9]. During replication, NS1 secreted as a hexamer enters the blood stream, wherein it participates in immune evasion. The detection of NS1 in serum can be utilized in diagnosis of acute DENV infection [10]. NS1 is a strong immunogen and during DENV infection gives rise to autoreactive antibodies targeting platelets and endothelial cells [11]. Even though the NS1 of ZIKV and DENV are ~50% identical at amino acid level and have conserved regions, ZIKV-specific epitopes have also been identified [12].

The adaptive immune response to primary ZIKV infection is similar to that of most viral infections with an initial IgM rise within 5–7 days post infection (dpi), followed by IgG response [13]. The IgM can remain detectable for several months in a person previously naïve for DENV infection, and the IgG response is thought to provide lifelong immunity; however, the long-term kinetics of ZIKV antibodies are as yet not well known. The current diagnostics includes reverse transciption PCR (RT-PCR), neutralization tests, enzyme-linked immunosorbent assay (ELISA) and immunofluorescence assays. Diagnosis based on RT-PCR is rather specific but provides a short diagnostic window, as ZIKV RNA in serum is usually detectable for only 5 to 8 dpi [13]. ZIKV RNA persists longer in whole blood, excretions and in pregnant women with an infected fetoplacental system [6, 14, 15]. Serodiagnostics have a significantly longer diagnostic window but are prone to antibody cross-reactivity between flavivirus species. This is especially problematic between DENV and ZIKV since both viruses are found in the ZIKV epidemic regions, with DENV seroprevalence in many areas exceeding 50%. However, commercial ELISAs for the detection of IgG/IgM antibodies specifically against ZIKV NS1 are available [16]. As a rule however, the IgM responses to ZIKV in and acute infection are lacking in DENV-immune persons [17].

**Fig 1:**
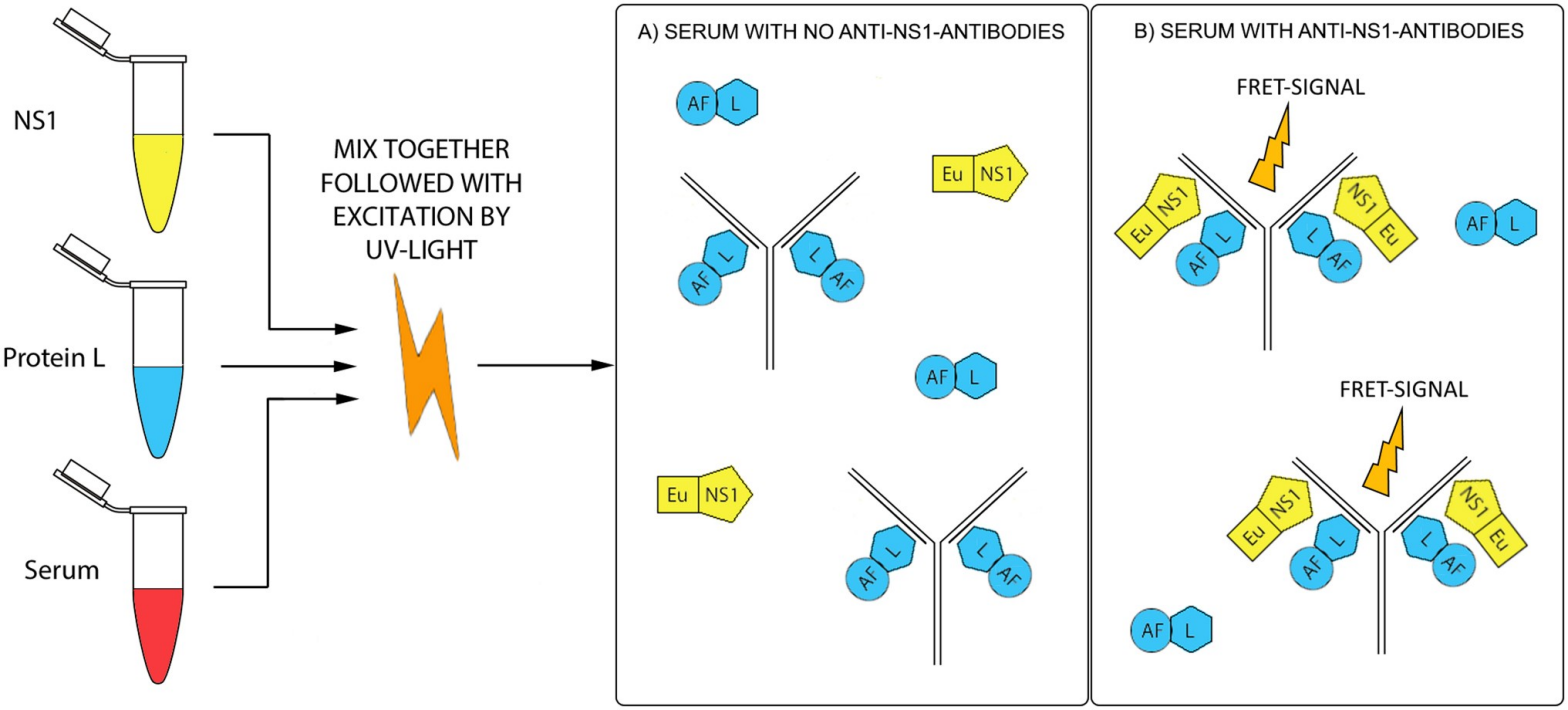
The LFRET assay principle. The Eu-labeled antigen (ZIKV NS1), Alexa-Fluor-labeled protein L and the serum sample are mixed together in a 384-well microplate. The former binds to the light chains of the antibodies, which if ZIKV-NS1-specific also bind the europium-labeled NS1 antigen. The mixture is exposed to UV light and the close proximity allows the donor and acceptor to elicit the FRET signal, which is measured.

We recently described a novel serodiagnostic approach for acute hantavirus disease based on time-resolved Förster resonance energy transfer (TR-FRET) [18]. For the FRET phenomenon to occur, two light-sensitive molecules (chromophores) must be in very close proximity (<<10 nm). When the donor chromophore is excited with ultraviolet radiation, the excitation energy is transmitted to the acceptor which then emits the energy. The excitation and the emission occur at distinct wavelengths, enabling separation of the signals. Chelated lanthanides such as Eu moreover exhibit a delay between the absorption and emission, allowing for time-resolved (TR) detection. The TR-FRET approach permits measurement from autofluorescent materials, including serum. The new approach utilizes the simultaneous binding of donor-labeled viral antigen and acceptor-labeled protein L, a bacterial superantigen that binds to immunoglobulin kappa light chain (κ-chain) [19]. When a clinical serum is mixed with the two antigens directly in solution, the possibly contained virus-specific antibodies bring the donor and acceptor to close proximity for the TR-FRET fluorescence to arise. We call this diagnostic principle LFRET, of which a schematic overview is shown in Fig 1.

The rapid spread and serious health implications of ZIKV highlight the need for reliable diagnostic tools. In this report, we demonstrate a method for ZIKV serodiagnosis using NS1 labelled with chelated Eu as the donor (excitation at 320 nm) and protein L labelled with AlexaFluor 647 as the acceptor (emission at 647 nm). We further evaluate the test by separate detection of the antibody classes and document the suitability of the new assay by comparison with a commercially available ELISA.

## Materials and methods

### Clinical samples and ethics statement

The ZIKV-positive samples had been sent for diagnosis at the Bernhard Nocht Institute for Tropical Medicine (Hamburg, Germany), and originate from Finnish or German travelers. The reference tests for the ZIKV-positive samples (n = 25) included Euroimmun ELISA, indirect immunofluorescence (IIF), neutralization test and RT-PCR and the results are summarized in Table 1. As seen, for several samples the ELISA and IIF results were inconsistent, most notably for IgM in 11/25 (36%). Therefore, we adopted strict reference criteria according to which a sample was considered seropositive only if the two reference results matched. Any samples with conflicting reference data were considered inconclusive and removed from analysis. This matter is discussed further in Results.

**Table 1:** Reference test results for ZIKV-positive samples.

Patient pseudonym	Sample #	Days from onset	ZIKV PCR^a^	ZIKV ELISA		ZIKV IIF		DENV IIF	
				IgG	IgM	IgG	IgM	IgG	IgM
Z1	1	65	neg	pos	neg	640	20	--	--
"	2	118	neg	pos	neg	160	20	--	--
"	6	308	--	pos	pos	1280	neg	--	--
"	8	407	--	pos	neg	320	neg	--	--
Z2	1	38	neg	pos	neg	5120	80	2560	neg
"	3	434	--	pos	neg	2560	neg	640	neg
Z3	1	35	neg	pos	pos	20480	80	1280	neg
"	3	426	--	pos	neg	5120	neg	2560	neg
Z4	1	16	pos	pos	pos	2560	1280	80	neg
"	3	52	--	pos	pos	1280	160	1280	neg
"	4	115	--	pos	neg	1280	40	80	n.T.
Z5	1	7	pos	pos	pos	5120	5120	80	neg
"	4	100	--	pos	neg	640	320	80	n.t.
Z6	1	19	pos	pos	pos	5120	40	5120	neg
Z7	1	11	pos	pos	pos	5120	1280	5120	neg
31	1	N/A	--	neg	pos	640	neg	--	--
188	1	N/A	--	neg	pos	20480	neg	--	--
229	1	N/A	--	pos	pos	pos	pos	--	--
354	1	N/A	--	pos	pos	pos	pos	--	--
595	1	N/A	--	pos	neg	pos	pos	80	20
775	1	N/A	--	neg	pos	neg	neg	--	--
864	1	N/A	pos	neg	pos	pos	pos	1280	neg
901	1	N/A	--	neg	pos	pos	pos	--	--
948	1	N/A	--	pos	pos	pos	pos	20480	80
956	1	N/A	--	neg	neg	pos	pos	--	--

^a^PCR from either urine or blood

The negative (no antibodies to flaviviruses (ZIKV, DENV, TBEV)) serum samples as well as those with antibodies to other flaviviruses (YFV recent vaccinees, acute-phase TBEV or DENV patients) had been sent to the Helsinki University Central Hospital Laboratory Service (HUSLAB, Virology and Immunology, Zoonosis Unit, Helsinki, Finland) for infectious disease serodiagnosis. The DENV samples (n = 38) were from Finnish travelers with acute illness, of which a subset (n = 34) were serotyped by partial sequencing of the NS5 gene. All four DENV serotypes were included (DENV1-DENV4; n = 16, n = 9, n = 3, n = 6 samples respectively) and most of the sera (n = 35) were also DENV NS1 antigen positive. The acute-phase TBEV samples (n = 20) came from Finnish patients and were IgM-positive in μ-capture ELISA [20] and also had elevated hemagglutination inhibition test titers. The YFV samples (n = 16) were IgG-positive post-vaccination controls (live attenuated 17D vaccine). These samples (n = 26) had been collected in Finland during 2009–2014, before the tropical ZIKV epidemic, and were assumed ZIKV seronegative. All samples in the negative pool that had an unknown ZIKV status were tested with EuroImmun IgM and IgG ELISA tests.

All patient samples and clinical data in the study were fully anonymized. The study was approved under research permit No. 32/2018 issued 09.03.2018 (latest renewal), §16, granted by the Hospital district of Helsinki and Uusimaa (Teaching and Research).

### Proteins

Recombinant protein L (Thermo Scientific, Pierce Protein Biology Products) was labeled with AlexaFluor 647 as described [21] to yield AF-protL. Recombinant ZIKV NS1 protein (Native Antigen Company, Oxfordshire, UK) was labeled according to the manufacturer’s instruction with europium (Eu), using the Quick-AIIAssay Eu-chelated protein labeling kit (BN Products & Services Oy) to yield Eu-NS1. The degree of labeling, as measured according to the manufacturers’ instructions, was 3–4 AF per molecule for protein L and 4.2–5.1 Eu per molecule for NS1.

### Test protocol

#### Immunoglobulin class-independent ZIKV LFRET

The protocol is modified from previous [21]. All dilutions were done in Tris-buffered saline (50 mM Tris-HCl, 150 mM NaCl) supplemented with 0,5% (w/v) BSA (TBS-BSA). The reactions were performed as follows: 5 μl of AF-protL (50 nM) and 5 μl of Eu-NS1 (20 nM) were mixed on 384-well microplate (ProxiPlate-384 Plus F; black 384-shallow-well microplate from PerkinElmer) and 10 μl of 1:50 diluted serum was added and mixed by pipetting. The final concentrations in the final 20 μl reaction volume were serum diluted 1:100, 25 nM AF-protL, and 10 nM Eu-NS1. After combining the constituents, the LFRET values were immediately measured with a Victor Wallac^2^ fluorometer (PerkinElmer), normalized and presented as integers (the LFRET count) as earlier [18, 22]. All measurements were performed in duplicate.

#### Class-specific ZIKV LFRET

To separate the TR-FRET counts induced by antibodies of distinct immunoglobulin classes, we developed two variants of the class-independent assay. For detection of acute infections the IgG antibodies were removed by mixing the serum 1:10 with GullSORB (Meridium Bioscience, USA) and centrifugation at 16,000 x g for 10 s. The supernatant was diluted 1:5 with TBS-BSA yielding a final dilution of 1:50, which was used in the assay described above. For IgG detection the IgM antibodies in the samples were inactivated as described [23]. Briefly, the sera were diluted 1:50 with TBS-BSA containing 5 mM dithiothreitol (TBS-DTT) and incubated for 2 h at +37 °C. The dilutions were then used as above.

## Results

### Immunoglobulin class-independent LFRET against ZIKV

**Fig 2:**
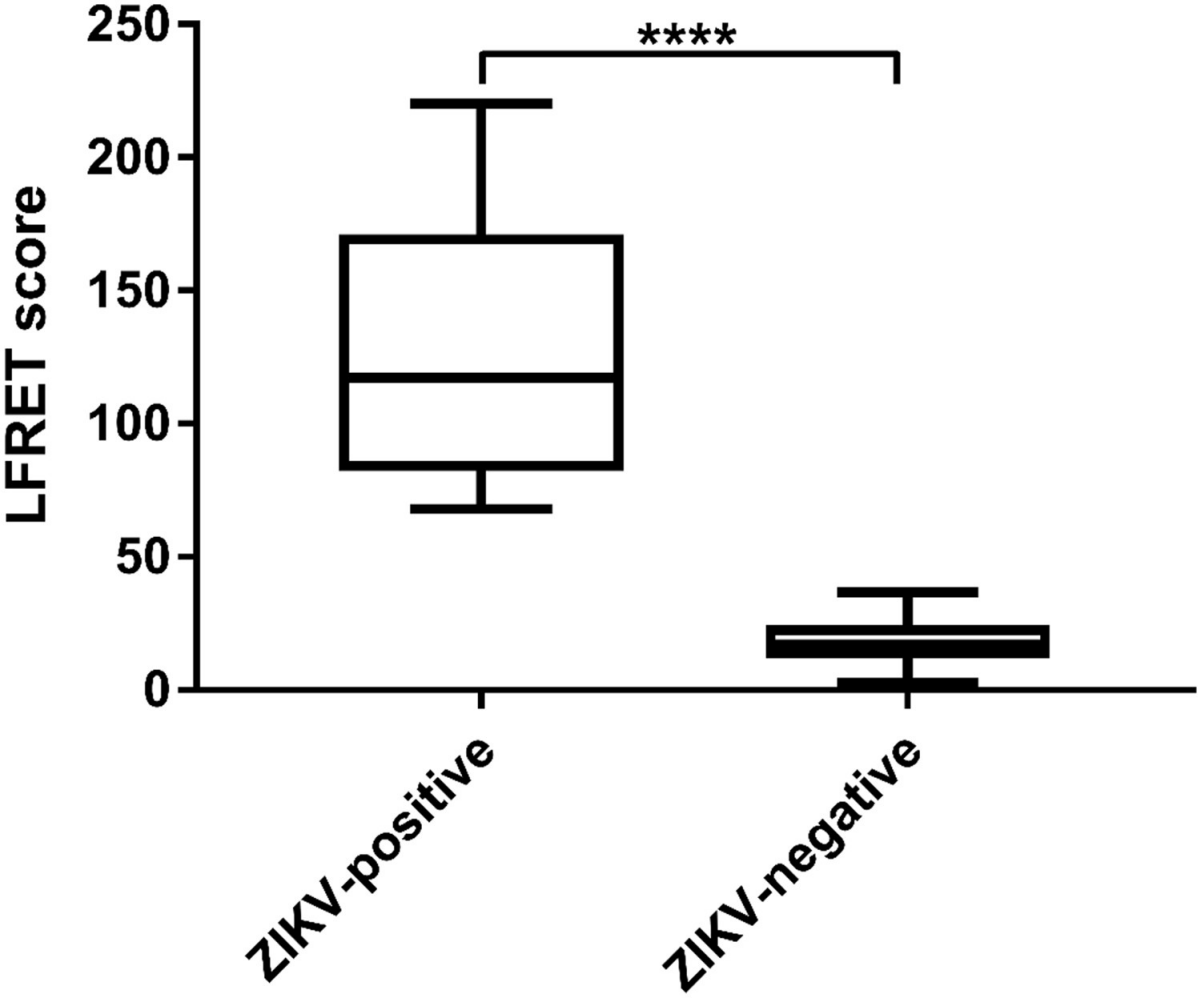
Class-independent LFRET scores for the first cohort of samples.

To set up the assay, we performed cross-titration experiments using pooled ZIKV seropositive and seronegative sera at various concentrations of Eu-labelled ZIKV-NS1 (Eu-NS1) and AlexaFluor-labeled protein L (AF-ProtL) and concluded the optimal concentrations of the reagents to be 20 nM and 50 nM, respectively. With these test parameters, we analyzed a cohort of ZIKV ELISA IgG- or IgM-positive samples (from patients Z1-Z7, n = 15) together with a panel of ZIKV seronegative samples (n = 24). The average LFRET count (AVG) for negative samples was 20.8 with a standard deviation (SD) of 12.8. From this data, we initially set a cut-off for positivity at AVG + 2.5*SD, LFRET count of 52.8. All of the 15 samples known to be positive had LFRET counts above this cut-off and their detailed results are shown in Fig 2.

**Fig 3:**
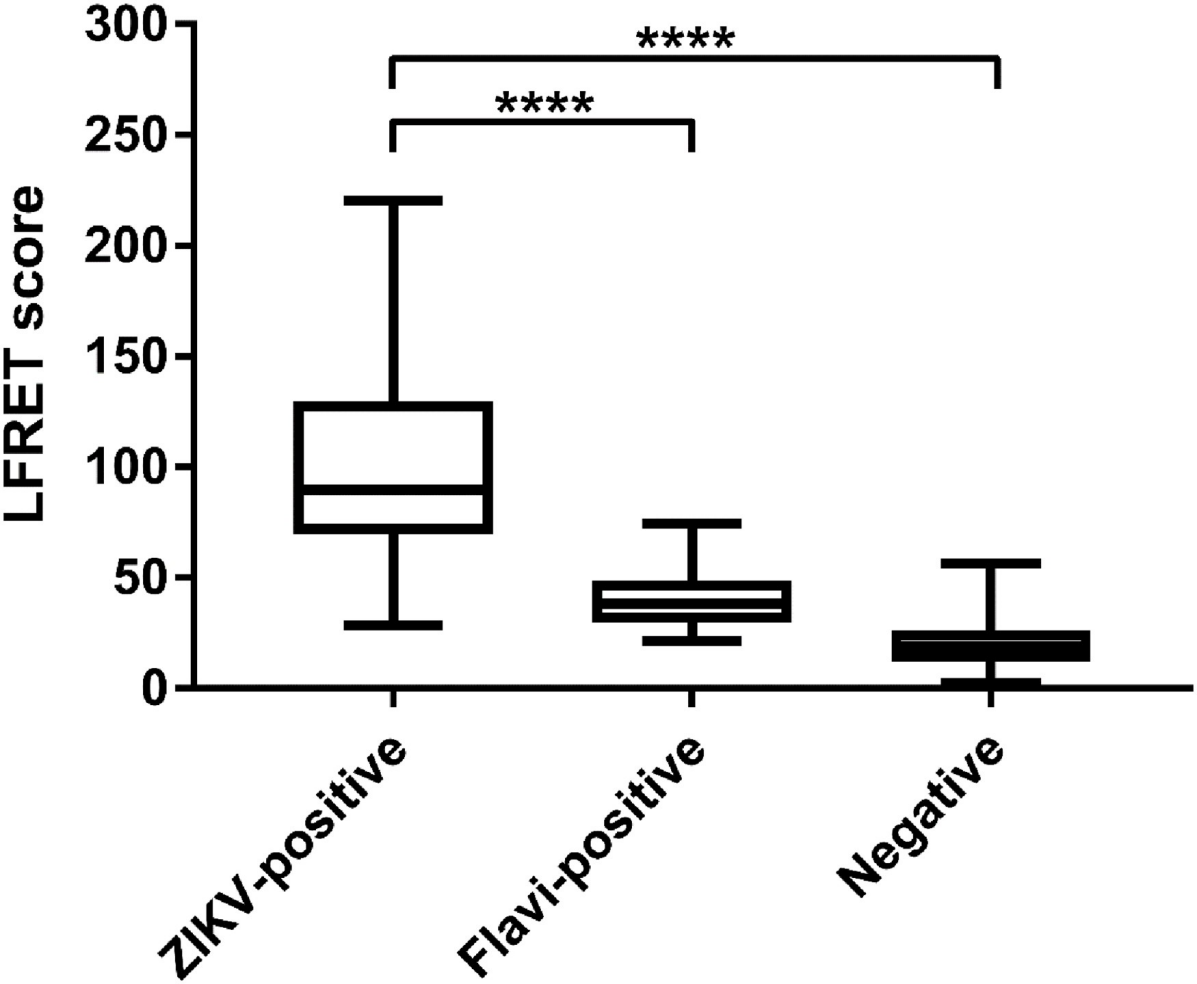
Class-independent LFRET scores measured from the full panel of ZIKV-positive, heterologous flavivirus-positive (DENV, YFV or TBEV) and flavivirus-negative samples.

Since flavivirus serodiagnostics are known to suffer from inter-species cross-reactivity, we then analyzed a panel of well-characterized sera representing acute infections with DENV (n = 38) and TBEV (n = 20) and YFV vaccinees (n = 16) as well as additional negative samples (n = 2) and cases of ZIKV (n = 10) with LFRET. The combined results (Fig 3) showed only minor cross-reactivity by the other flaviviruses with ZIKV. After reviewing the data, we adjusted the cut-off value for positivity to LFRET count 57, corresponding to AVG + 2.8*SD. Including all sample categories the assay had a sensitivity of 92%. The specificity in the heterologous flavivirus seropositive samples was 87% (88% for DENV and 87% for YFV), and 100% among flavivirus seronegative samples. The test outcomes of all sample types are presented in Table 2 and the complete result set is presented in S1 Table.

**Table 2:** Class-independent LFRET test outcomes in different sample categories.

	ZIKV-positive (n = 25)	DENV-positive (n = 38)	YFV-positive (n = 16)	TBEV-positive (n = 20)	Negative (n = 26)
LFRET- positive	92% *(n = 23)*	12% *(n = 5)*	13% *(n = 2)*	0% *(n = 0)*	0% *(n = 0)*
LFRET- negative	8% *(n = 2)*	88% *(n = 33)*	87% *(n = 14)*	100% *(n = 20)*	100% *(n = 26)*

### Class-specific LFRET assays

The LFRET assay is based on the simultaneous binding of protein L and the antigen to the same antibody, and the LFRET counts measured from human serum can be derived from any Ig class. As such, further analysis of the samples was required to see whether the LFRET test could be utilized to distinguish between signals induced by IgM and IgG. To measure the counts induced by antibodies other than IgG, ie. to detect acute infection, we depleted IgG from the samples using GullSORB reagent. Following IgG depletion, the ZIKV-specific LFRET signals from serum are induced by IgM and IgA (also to a lesser degree IgE). To determine immunity by measuring ZIKV-specific IgG, we inactivated IgM antibodies from the samples using 5 mM DTT. Given that IgA antibodies are more resistant to DTT than IgM and not fully inactivated in this protocol [24], this variant can also measure ZIKV-specific IgA. IgE antibodies are rendered inactive with DTT concentrations over 2 mM and hence undetectable [25]. The IgG depletion variant will be referred to as acute-LFRET and the IgM inactivation variant as immunity-LFRET.

To obtain baseline values for negative samples, we tested a cohort of negative samples with both class-specific variants. Similarly to class-independent LFRET, we calculated a cut-off for positivity at AVG + 2.5*SD yielding cut-offs for the acute and immunity assays at 62 and 65, respectively. After the preliminary runs we tested all of the remaining samples in both assay variants and the complete result set is presented in S1 Table.

**Fig 4:**
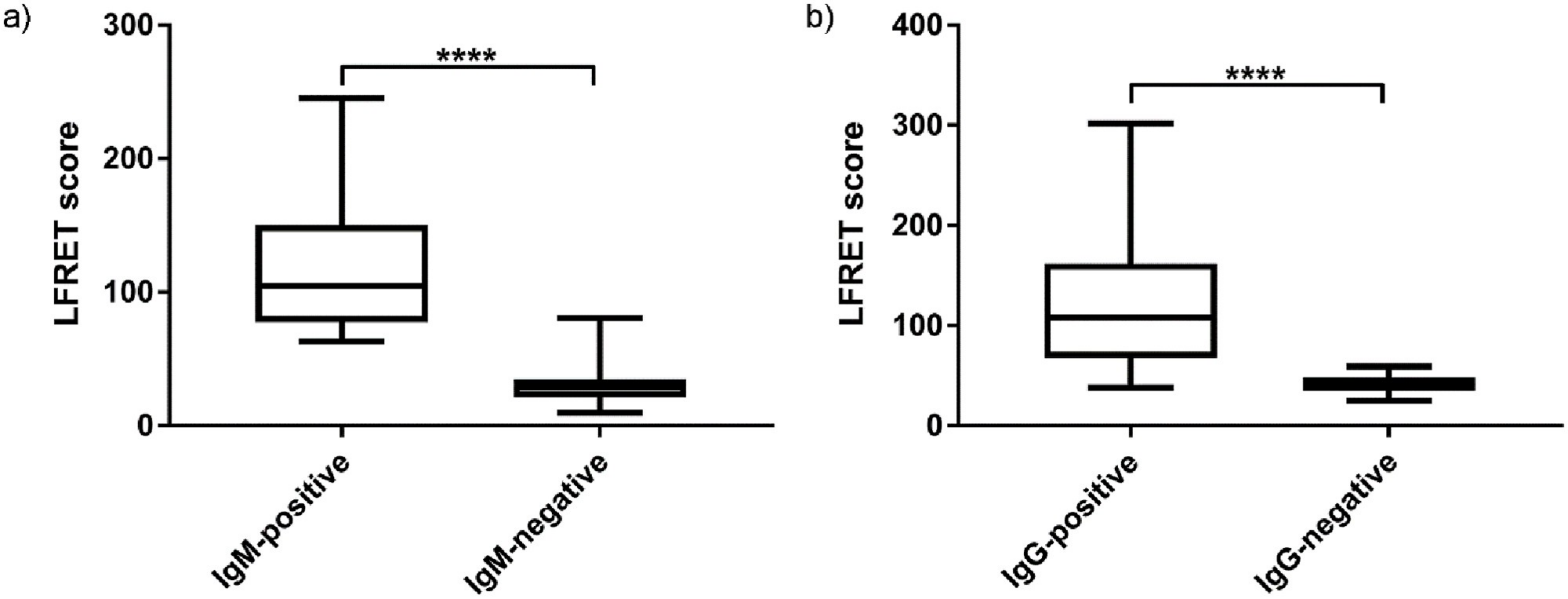
a) Acute-LFRET scores of IgM-positive and -negative samples b) Immunity-LFRET scores of IgG-positive and -negative samples.

As stated in Materials and methods, due to the inconsistencies between the reference methods (Table 1) we initially deemed the samples seropositive excusively when the two reference methods agreed. We classified any samples with divergent ELISA and IIF results (n = 11 and n = 5 for IgM and IgG, respectively) as inconclusive and removed them from statistical analysis. Some of the ZIKV-negative samples were omitted in acute-LFRET (n = 1) and immunity-LFRET (n = 17) due to shortage of the original material. Based on these criteria the test panel contained 113 samples for acute-LFRET and 103 samples for immunity-LFRET. The class-specific LFRET results of this cohort are presented in Fig 4 and Table 3. In both acute-FRET and immunity-LFRET, cross-reactivity with other flaviviruses played only a minor role, as evidenced by the respective specificities of 96% and 100%, at sensitivities of 100% and 95%. Interestingly, both of the class-specific assays showed significantly higher specificity than the class-independent LFRET (specificity 88%).

**Table 3:** Test outcomes in class-specific LFRET tests compared with the reference tests.

a)	Anti-ZIKV-IgM-positive (n = 11)	Anti-ZIKV-IgM-negative (n = 102)	b)	Anti-ZIKV-IgG-positive (n = 19)	Anti-ZIKV-IgG-negative (n = 84)
Acute-LFRET positive	100% *(n = 11)*	4% *(n = 4)*	Immunity-LFRET positive	95% *(n = 18)*	0% *(n = 0)*
Acute-LFRET negative	0% *(n = 0)*	96% *(n = 98)*	Immunity-LFRET negative	5% *(n = 1)*	100% *(n = 84)*

a) Acute-LFRET outcomes vs. IgM-reference tests

b) Immunity-LFRET outcomes vs. IgG-reference tests

We next carried out a assay evaluation including all samples positive in either ELISA or IIF, or both. The acute- and immunity-LFRET results based on these criteria are presented in Table 4. The inclusion of these looser criteria did not alter the sensitivity or specificity (100% and 96%) of the acute-LFRET. On the other hand, while the specificity of the immunity-LFRET remained at 100%, its sensitivity decreased to 83%.

**Table 4:** Test outcomes when using non-rigorous sample inclusion criteria.

a)	Anti-ZIKV-IgM-positive (n = 22)	Anti-ZIKV-IgM-negative (n = 102)	b)	Anti-ZIKV-IgG-positive (n = 24)	Anti-ZIKV-IgG-negative (n = 84)
Acute-LFRET positive	100% *(n = 22)*	4% *(n = 4)*	Immunity-LFRET positive	83% *(n = 20)*	0% *(n = 0)*
Acute-LFRET negative	0% *(n = 0)*	96% *(n = 98)*	Immunity-LFRET negative	17% *(n = 4)*	100% *(n = 84)*

a) Acute-LFRET outcomes vs. IgM-reference tests

b) Immunity-LFRET outcomes vs. IgG-reference tests

### Comparison of LFRET with a commercial ELISA

We compared the performance of the new assay to a commercially available ZIKV NS1-based IgM and IgG ELISAs (Euroimmun, Germany) [16] using a subset of the samples. The subset consisted of all the ZIKV-positives (by any reference test, n = 25) as well as some DENV- (n = 23) and YFV-positives (n = 10) and all samples that had cross-reacted in any of the three LFRET assay variants.

**Fig 5:**
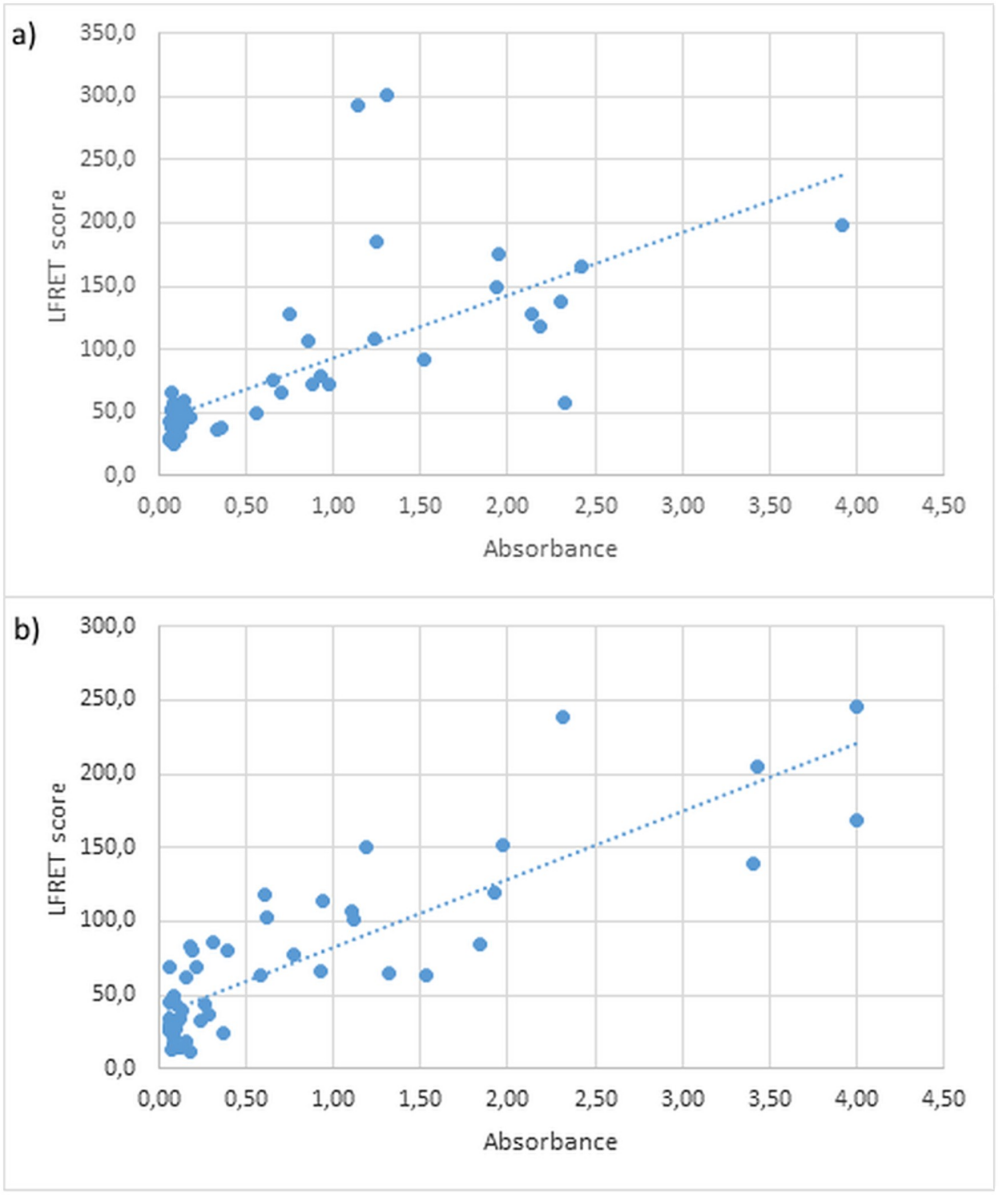
Euroimmun ELISA vs. class-specific LFRET. a) Optical density values in Euroimmun IgM ELISA compared to the acute-LFRET scores of the same samples; b) OD values in Euroimmun IgG ELISA test compared to the immunity-LFRET score.

To analyze the results we compared the optical density (OD) values measured in the ELISAs with the LFRET scores for each sample (Fig 5). These results are listed in S2 and S3 Tables. There was a moderate correlation between the immunity-LFRET scores and the IgG ELISA ODs (correlation coefficient r = 0.68). However, the acute-LFRET scores correlated strongly with the corresponding IgM ELISA ODs (r = 0.85). In Student’s t-test both correlations were highly significant (p<0.001).

Following the observation that the LFRET and ELISA assays were correlated in terms of the measured values, we then compared their binary outcomes (positive / negative). The IgM and IgG outcome comparison is presented in Table 5. Despite the overall good correlation, the LFRET and ELISA outcomes of several samples diverged, especially for IgM, and these results are listed in Table 6. In the majority of such cases (9/12), the LFRET and IIF were positive while the ELISA was negative.

**Table 5:** Comparison of the outcomes of the Euroimmun ELISA test with class-specific LFRET test outcomes.

a)	Euroimmun IgG ELISA positive	Euroimmun IgG ELISA negative	b)	Euroimmun IgM ELISA positive	Euroimmun IgM ELISA negative
Immunity- LFRET positive	17	3	Acute-LFRET positive	15	11
Immunity-LFRET negative	1	33	Acute-LFRET negative	0	26

a: Outcome of IgG ELISA vs. immunity-LFRET.

b: Outcome of IgM ELISA vs. acute-LFRET.

**Table 6:** The samples with inconsistent results between Euroimmun ELISA and class-specific LFRET assay.

Pseudonym	Sample #	IgM		IgG		Inconsistent antibody class^a^	LFRET result consistent with IIF^b^
		ELISA	LFRET	ELISA	LFRET		
Z1	1	-	+	+	+	IgM	Yes
"	2	-	+	+	+	IgM	Yes
"	8	-	+	+	+	IgM	No
Z2	1	-	+	+	+	IgM	Yes
"	3	-	+	+	+	IgM	No
Z3	1	+	+	-	+	IgG	Yes
Z4	4	-	+	+	+	IgM	Yes
Z5	1	+	+	+	-	IgG	No
"	4	-	+	+	+	IgM	Yes
188	1	+	+	-	+	IgG	Yes
595	1	-	+	+	+	IgM	Yes
956	1	-	+	-	+	IM and IgG	Yes

^a^The class of antibody that has an ELISA result inconsistent with the LFRET result.

^b^Is the LFRET result consistent with the IIF reference results.

## Discussion

Herein we describe a simple TR-FRET-based homogenous serological assay for ZIKV infection. We examined a panel of 25 ZIKV-antibody positive travelers’ samples, a cohort of samples with another clinical flaviviral infection or recent vaccination and seronegatives. We developed three variants of the assay and showed a diagnostic specificity and sensitivity of higher than 90% in all variants.

Cross-reactivity has traditionally been a major problem in the serodiagnosis of flaviviruses since the different species are antigenically closely related. Many flaviviruses also co-circulate and pre-existing immunity towards at least one member of the genus is frequently a confounding factor in serological diagnosis. Because of this, we included several samples with heterologous flaviviral infections to the test panel. DENV is antigenically very similar to ZIKV and widely endemic with high seroprevalence and it is an important source of cross-reactivity. In our panel, the samples from acute primary DENV infections gave rise to some false positive reactivities in the class-independent assay, which in the class-specific assays however remained absent. Similarly, YFV also elicited some false reactivity in the class-independent assay but none in the class-specific variants. Overall, cross-reactivity did not significantly hamper the differentiation of ZIKV-specific antibodies from other flaviviruses. Rapid assessment of ZIKV-positive sera from DENV-endemic areas in Brazil showed high specificity but lowered sensitivity. The same phenomenon has been observer for other serological tests [18] and further experiments with well characterized positive and negative sera form DENV-endemic areas are needed to assess the usability of this test in DENV-immune individuals.

As noted, in the panel of ZIKV positive samples there were many instances where the reference tests ELISA and IIF were inharmonious. As no “gold standard” exists in ZIKV serodiagnostics, these two reference tests were employed to corroborate each other. Accordingly, a sample was considered positive only if both of the two were positive, and the remaining samples were excluded from evaluation. This diminished the number of samples especially for IgM (n = 11), and reduced the statistical power. Such strictness of inclusion criteria we considered essential for the reliability of the reference tests. However, for completeness we also used an approach where the inclusion criteria was a positive result in either reference test. Even with this less rigorous sample selection, the assays showed high specificity and sensitivity.

Unlike in the classical serodiagnostics such as ELISA or IIF, in which a single antibody class is detected at a time, the LFRET follows a different strategy. In class-independent LFRET all antibody isotypes are detected simultaneously, whereas in the acute and immunity variants one immunoglobulin class at a time is removed and those that remain are detected. Therefore, for example, any specific IgA or IgE, which are often otherwise ignored, will be included in the LFRET result. This will permit the detection of un-orthodox antibody responses (e.g. IgA-only), and potentially allow for diagnosis with increased sensitivity.

To gauge the performance of LFRET further, we compared the results from the class-specific assays against Euroimmun ELISA, a commercial kit based on the same antigen, ZIKV NS1. As stated in Results, the numerical measurements of the assays correlated strongly; however, the binary outcomes often did not. Looking into these data it was evident that in most cases the ELISA was negative, while both LFRET and IIF were positive. There are some indications of this ELISA having a somewhat low sensitivity, e.g. as compared to the combination of a CDC-approved IgM antibody capture ELISA and plaque reduction neutralization test [26], and our data seems to show parallel findings.

The present is the second application of the LFRET approach, the first being developed for hantavirus disease [18]. Similarly to latter, the ZIKV assay had a diagnostic specificity and sensitivity of >95%. These findings suggest that the wash-free LFRET assay adapted to a point-of-care setting has potential for assisting in clinical decision-making.

## Supporting information

S1 Table The complete LFRET result listing. This table contains the LFRET test results for all samples in all of the assay variants.
(XLSX)

S2 Table IgM ELISA and acute-LFRET. This table contains a comparison of the absorbance values and test outcomes of Euroimmun IgM ELISA against the acute-LFRET results.
(XLSX)

S3 Table IgG ELISA and immunity-LFRET. This table contains a comparison of the absorbance values and test outcomes of Euroimmun IgG ELISA against the immunity-LFRET results.
(XLSX)
